# Complimentary Letters

**Published:** 1851-07

**Authors:** 


*Complimentary Letters.*—We have failed to reply to many letters received, conveying much of our best reward in their commendatory terms of our efforts to accomplish the object of the Journal, and we beg to return to each and all, our sincere thanks for their grateful encouragement, and trust we may be able ever to retain such valuable opinions. Such letters, coming from men of the highest standing and abilities, induce us to believe, that we have not entirely failed in our purpose, of producing a periodical, which has met the wants of the profession.

This No. completes the first volume, and we shall enter upon the second with renewed efforts and encouragement.

